# Improved Positron Emission Tomography Quantification: Evaluation of a Maximum-Likelihood Scatter Scaling Algorithm

**DOI:** 10.3390/diagnostics14111075

**Published:** 2024-05-22

**Authors:** Nanna Overbeck, Sahar Ahangari, Maurizio Conti, Vladimir Panin, Aleena Azam, Sorel Kurbegovic, Andreas Kjær, Liselotte Højgaard, Kirsten Korsholm, Barbara Malene Fischer, Flemming Littrup Andersen, Thomas Lund Andersen

**Affiliations:** 1 Department of Clinical Physiology and Nuclear Medicine, Copenhagen University Hospital—Rigshospitalet, 2100 Copenhagen, Denmark; nanna.overbeck.petersen.01@regionh.dk (N.O.); andreas.kjaer@regionh.dk (A.K.); liselotte.hoejgaard@regionh.dk (L.H.); kirsten.korsholm.01@regionh.dk (K.K.); barbara.malene.fischer@regionh.dk (B.M.F.); flemming.andersen@regionh.dk (F.L.A.); 2 Siemens Medical Solutions Inc., Knoxville, TN 37932, USA; maurizioconti@siemens-healthineers.com (M.C.); vladimir.panin@siemens-healthineers.com (V.P.); 3 Cluster for Molecular Imaging, Copenhagen University Hospital—Rigshospitalet, 2100 Copenhagen, Denmark; aleena.azam@regionh.dk (A.A.); sorel.kurbegovic@regionh.dk (S.K.); 4 Department of Biomedical Sciences, University of Copenhagen, 2100 Copenhagen, Denmark; 5 Department of Neurosurgery, Neuroscience Center, Copenhagen University Hospital—Rigshospitalet, 2100 Copenhagen, Denmark

**Keywords:** scatter scaling, scatter correction, PET/MRI, maximum likelihood, halo artifacts

## Abstract

Incorrect scatter scaling of positron emission tomography (PET) images can lead to halo artifacts, quantitative bias, or reconstruction failure. Tail-fitted scatter scaling (TFSS) possesses performance limitations in multiple cases. This study aims to investigate a novel method for scatter scaling: maximum-likelihood scatter scaling (MLSS) in scenarios where TFSS tends to induce artifacts or are observed to cause reconstruction abortion. [^68^Ga]Ga-RGD PET scans of nine patients were included in cohort 1 in the scope of investigating the reduction of halo artifacts relative to the scatter estimation method. PET scans of 30 patients administrated with [^68^Ga]Ga-uPAR were included in cohort 2, used for an evaluation of the robustness of MLSS in cases where TFSS-integrated reconstructions are observed to fail. A visual inspection of MLSS-corrected images scored higher than TFSS-corrected reconstructions of cohort 1. The quantitative investigation near the bladder showed a relative difference in tracer uptake of up to 94.7%. A reconstruction of scans included in cohort 2 resulted in failure in 23 cases when TFSS was used. The lesion uptake values of cohort 2 showed no significant difference. MLSS is suggested as an alternative scatter-scaling method relative to TFSS with the aim of reducing halo artifacts and a robust reconstruction process.

## 1. Introduction

The combination of positron emission tomography (PET) with either computed tomography (CT) or magnetic resonance imaging (MRI) can provide information about cancer development, progression, and localization [1]. Accurate scatter correction of PET data is a necessity to obtain image quantification that improves the diagnostic quality of the resulting images [2,3,4,5,6,7,8,9]. Widely used scatter-scaling algorithms include tail-fitted scatter scaling (TFSS) and absolute scatter correction (ABS), both of which are based on a scatter distribution estimate calculated from a single scatter simulation (SSS) [9,10,11,12]. Scatter scaling by the use of TFSS subsequently rescales the scatter estimate and prompt gamma (PG) distribution following the tails of the scatter distributions based on the sinograms. ABS is based on the scatter distribution applied directly from SSS, which makes it prone to an underestimation of scatter due to the exclusion of multiple scatter events [13]. Furthermore, ABS does not account for scatter from outside the field of view (FOV) or include PG correction. TFSS may cause overcorrection of scatter typically observed in areas of high tissue contrast/high tracer accumulation, such as near the urinary system, including the kidneys and bladder region, leading to areas of so-called halo artifacts [14,15]. Furthermore, TFSS may also lead to the overcorrection of scatter in cases of poor count statistics in the sinogram tail region. This is observed for receptor-based tracers and/or tracers with high specific uptake, such as ^68^Ga-labeled prostate-specific membrane ([^68^Ga]Ga-PSMA-11) [10,13]. Examples of tracers that possess high specificity are ^68^Ga-NODAGA-E[c(RGDyK)]2 ([^68^Ga]Ga-RGD) [16] and ^68^Ga-urokinase-Plasminogen-Activator-Receptor ([^68^Ga]Ga-uPAR) [17,18,19]. [^68^Ga]Ga-RGD targets angiogenesis by use of the integrin expression of αvβ3 [16]. Information regarding the [^68^Ga]Ga-RGD tracer is described by Oxboel et al. (2014) [20]. [^68^Ga]Ga-uPAR can, e.g., be utilized in investigations of glioblastomas and expresses proteases regarding proteolytic degradation of the extracellular matrix [17,18,19]. Tracers that facilitate specific processes within the body can provide biological information about the specific patient and support the individualization of cancer diagnostics [21]. The specificity of the tracer leads to poorer count statistics outside of the emission support used in TFSS, subsequently inducing a risk of decreasing the accuracy of TFSS scatter estimation.

A PG of energy of 1077 keV will be emitted simultaneously with the positron emission during the decay of ^68^Ga. The PG emission occurs with a branching ratio of approximately 1.2%. The branching of ^68^Ga might influence the scatter ratio if scattered PG is detected as a coincidence with an annihilating photon. The unscattered PG rays are above the energy window of the PET scanners (e.g., 430 to 610 keV for the Siemens Biograph mMR). Thus, they are not directly seen as the cause of halo artifacts [14]. It has been shown by Noto et al. (2017) that PG correction has a limited influence on the halo artifact concerning ^68^Ga-PET imaging [22]. The halo artifacts have been studied not only for a range of tracers and PET systems, such as [^68^Ga]Ga-PSMA-11 for PET/MRI [6,14,22] and PET/CT [7], but also with the administration of 2-deoxy-2-[^18^F]fluoro-D-glucose ([^18^F]FDG) for PET/CT [15], suggesting the artifact to be a common issue for a range of tracers and modalities. Halo artifacts are frequently seen in the pelvic area of reconstructed patient images due to the proximity to the bladder [4,15,22]. This is critical for patients with lesions located within the pelvic region, such as cases of cervical cancer or prostate cancer. Cervical cancer is the fourth-most-frequent type of cancer in women [23], and the appearance of halo artifacts due to incorrect scatter scaling during the reconstruction can lead to inaccurate clinical decision making, inability to locate metastases, and incorrect determination of the total circumference of tumors [22,24]. The halo artifact is commonly seen, consequently, in the use of the TFSS because the fitting and scaling of the scatter is applied over high contrast intensity values [10,13,25]. The scatter is scaled based on the sinogram tails derived from outside the CT-based attenuation correction mask, implying that, in cases of very low physiological uptake values inside the object, the scatter, as determined by the sinogram tails outside the mask, can become poorly determined and even unpredictable. This is most prominent in cases of brain-scan reconstructions with low background uptake values, i.e., in highly specific tracers. In cases of low-count statistics, ABS may be used as a fallback method due to low-count statistics in the tail region. The disadvantage of using ABS is the underestimation of the scatter leads to incorrect uptake values in the final reconstruction. Thus, it should not be used for quantitative tasks [13]. It is observed in clinical routines that low count statistics in the tail regions can cause a TFSS-corrected reconstruction process to abort due to non-negativity constraints.

To overcome the challenges of TFSS, Bal et al. (2018) have developed a maximum-likelihood scatter simulation (MLSS) algorithm based on the work of Panin et al. (2012) and Rezaei et al. (2017) [2,8,11]. MLSS utilizes the CT-based attenuation map and an image estimate in a computation process in which both the activity image and scatter sinogram are simultaneously updated through four iterations. At the PET/MRI, this will be addressed by MRI-derived attenuation maps. In this study, we use AI-derived synthetic CT based on DIXON Vibe sequences, as described by Ahangari et al. (2021), Ahangari et al. (2022b), and Ladefoged et al. (2019) [26,27,28]. In the process of computing the scatter estimate, MLSS allows for negative values in the scatter-corrected images and during the initial iterations. In addition, the difference between the MLSS and TFSS methods is the use of the entire emission sinogram to scale the scatter rather than performing the fitting in a limited region of the FOV. A preliminary evaluation of MLSS by Bal et. al. (2021) provides results implying that MLSS is acceptable for substituting the clinical standard within the reconstruction procedure [13].

This study aims to evaluate the performance of the MLSS algorithm compared to the state-of-the-art scaling method, TFSS, using either [^68^Ga]Ga-RGD cervical cancer PET/MRI scans or [^68^Ga]Ga-uPAR brain PET/MRI scans. Since no ground truth is known, the methods are compared by evaluating the uptake values relative to each other. The hypothesis of the used scatter-scaling methods is that MLSS and TFSS are expected to provide similar uptake estimates, except in the situations where TFSS might introduce halo artifacts or reconstruction abortion. It is investigated whether MLSS is a robust alternative when the clinical standard, TFSS, tends to fail due to, e.g., low background uptake, which is observed during brain scans with tracers with high specificity.

## 2. Materials and Methods

### 2.1. Image Acquisition

This study includes two cohorts of patients: patients with cervical cancer and patients with suspected glioma. The cohorts are used for the investigation of scatter-scaling differences using TFSS and MLSS during the image reconstructions.

Examinations for both patient cohorts were carried out on a hybrid PET/MRI system (Biograph mMR, Siemens Healthineers AG, Erlangen, Germany).

Cohort 1 consists of nine female patients with primary cervical cancer. The mean age of the patients is 57 ± 14 years old (range: 29–74 years old). Further information about patient weight, height, and BMI is overviewed in Table 1. The patients were injected with an average of 198.6 MBq (range: 172–210 MBq) [^68^Ga]Ga-RGD and rested for approximately 30 min before scan acquisition. The patients were scanned in a feet-first position for 20 min at a single bed position covering only the pelvic area. The hands were positioned on the chest outside FOV. The examinations were performed in the period from December 2019 to August 2021. Additional information regarding the patient cohort can be found in the study by Ahangari et al. (2022a), from which 9 out of 10 patients are included in this paper [29]. One patient was excluded due to missing PET data. Patients have given written informed consent, and the Regional Ethics Committee has approved the study (ref. no. H-18042903) [29].

Attenuation correction maps were calculated from artificial intelligence (AI) deep-learning inferred synthetic CT (sCT), as described by Ahangari et al. (2021) and Ahangari et al. (2022b) [26,27].

Thirty patients (15 females and 15 males) with a suspected glioma were included in cohort 2. The age of the patients was 59 ± 18 years old (range: 23–89 years old). The cohort includes patients scanned during the period from March 2017 to June 2022. Patients have given written informed consent, and the Regional Ethics Committee has approved the study (ref. no. VEK H-16035303).

The patients were positioned head first and arms along the body. They were injected bedside with an average of 200 MBq ± 26.8 MBq (range: 83–220 MBq) [^68^Ga]Ga-uPAR and scanned for 60 min. The scan covered the full head at a single bed position. A deep-learning AI-inferred attenuation map (DeepUTE) was generated based on an ultra-short echo-time (UTE) sequence. The generation of the attenuation map is described by Ladefoged et al. (2019) [28].

All patient data were treated fully anonymously in accordance with the European General Data Protection Regulation (GDPR).

### 2.2. Image Reconstruction

Scans from both cohorts were reconstructed offline with e7tools version VE11P for cohort 1 and VA20 for cohort 2 (Siemens Medical Solutions Inc., Knoxville, TN, USA) using a 3D Ordinary Poisson Ordered Subset Expectation Maximization (OP-OSEM) [30] with 3 iterations and 21 subsets. A Gaussian post-filtering was performed with 4 mm FWHM, resulting in a matrix of 344 × 344 (2.1 × 2.1 mm^3^) and a 2 mm slice thickness. 

Two scatter-scaling algorithms were applied and integrated with four iterations of the SSS algorithm, including a standard TFSS and an MLSS both implemented as a Siemens MATLAB prototype (Siemens Medical Solutions, Knoxville, TN, USA) integrated with e7tools. The first iteration scales the scatter based on an uncorrected image for both scatter-scaling methods. Scatter scaling by the use of TFSS is through scaling the tails to the net trues. MLSS-integrated scatter scaling elaborates the scatter relative to the scaling factors and the corresponding image update within each iteration. MLSS scaling factors are improved by the definition of the image support, which includes setting voxels outside the emission boundary to 0. This is optimized by making an initial image mask and finding the mean value of the boundary voxels. If a value outside the mask is greater than the mean value of the boundary voxels, the image mask is updated. Details of the process of TFSS and MLSS are described by Bal et al. (2019) [10].

Corrections made throughout reconstruction were normalization, random smoothing, xy-smoothing, z-smoothing, dead time, measured attenuation, 3D scatter, decay, frame-length, PG, and bed removal corrections. The coincidence-window width was 5.86 ns, and the energy window was defined to be within 430–610 keV.

TFSS- and MLSS-corrected reconstructions are termed TFSS reconstructions or MLSS reconstructions for simplicity throughout this paper.

### 2.3. Data Analysis

#### 2.3.1. Cohort 1

The reconstructions were analyzed qualitatively and quantitatively. The qualitative assessment was performed by an experienced nuclear medicine physician, using a 3-point score (1 = low image quality, 2 = acceptable image quality, and 3 = high image quality). Low image quality included the presence of halo artifacts to a degree that influences clinical decision making. High image quality includes image reconstruction without the visible appearance of halo artifacts. The evaluation was performed blinded with respect to the scatter-scaling method of use. The mean image-quality score was reported for each scatter method. 

**Figure 1:**
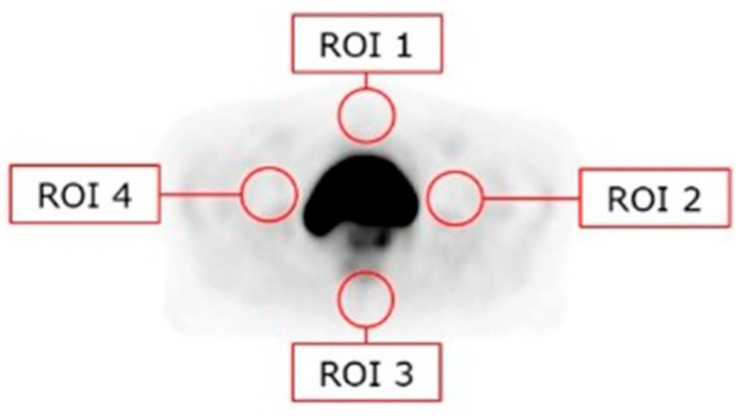
The red circles indicate the placement of possible ROIs used for the quantitative evaluation of the reconstructed images.

For the quantitative assessments, circular regions of interest (ROIs) were placed anteriorly, posteriorly, and in both lateral directions relative to the bladder. ROI had a diameter of approximately 5 cm with respect to the anatomy of the individual patient. These were termed ROI 1, 2, 3, and 4, respectively, as illustrated in Figure 1. Furthermore, the lesions were evaluated by an isocontour of 40% threshold of the maximum uptake. For each ROI, the mean uptake value was extracted in kBq/mL, and the analysis was performed by use of syngo.via imaging software (version VB60A, Siemens Healthineers AG, Erlangen, Germany). The uptake values of the reconstructions are compared by evaluating the relative difference of the ROI mean standardized uptake values (SUVs) calculated as (MLSS-TFSS)/MLSS × 100%.

#### 2.3.2. Cohort 2

The analysis was performed by registering the number of successful reconstructions for each of the scatter-scaling methods. Furthermore, the scans were divided into two groups by “positive” or “negative” visual uptake within the tumor. For the “positive” group, the lesion uptake was found using an isocontour of 40% threshold of the maximum uptake. An ROI was placed in the contra-lateral position for patients with lesion uptake to evaluate the background uptake of the brain. Patients of the “negative” group were analyzed by an ROI within the brain to investigate the general uptake. The ROI was placed, avoiding covering the skull and the center of the ventricles. The ROIs of both groups had a diameter of 7.2 cm ± 0.03 cm and were analyzed using syngo.via imaging software (Version VB60A, Siemens Healthineers AG, Erlangen, Germany), where the mean value within the ROI was noted in the unit of kBq/mL.

## 3. Results

### 3.1. Cohort 1

**Figure 2:**
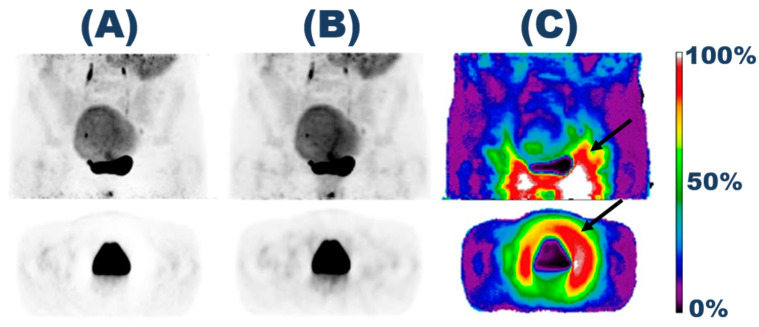
Visualization of the reconstructed scan of a patient of cohort 1. The figure includes reconstructions based on (**A**) TFSS and (**B**) MLSS. These are visualized along with (**C**) the relative difference with MLSS as a reference. The black arrows highlight the area of the halo artifact.

Figure 2 illustrates a patient scan reconstruction using (A) TFSS, (B) MLSS, and (C) the calculated relative difference given in percent. The findings visualized a difference between the two scatter-scaling methods, especially in the bladder region. Figure 2A shows a visible halo artifact around the bladder, which is not present for the MLSS reconstruction (Figure 2B). 

The qualitative evaluation of the reconstructed images was assessed by a score ranging from one to three, which correspond to low, acceptable, and high image quality, respectively. The resulting scores are shown in Table 2. MLSS reconstructions had an average score of 2.89, whereas TFSS reconstructions had an average score of 2. Thus, the reconstructions by use of MLSS were of high image quality, whereas TFSS showed acceptable image quality. 

**Figure 3:**
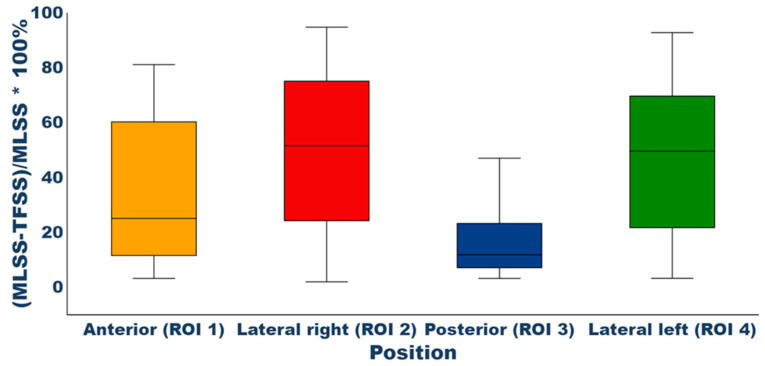
Boxplot of the relative difference between MLSS and TFSS in percent. The figure illustrates a boxplot for each position of the four ROIs based on all patients within cohort 1.

The quantitative investigation of the tracer uptake in the area in proximity to the bladder showed differences between the reconstructed images by use of the two scatter-scaling methods. The maximum differences found within the mean uptake value of the ROIs positioned lateral relative to the bladder were found to be of the magnitude of 94.7% and 92.8%, respectively. Figure 3 illustrates an overview of the evaluation of the ROIs positioned in proximity to the bladder. ROI 1 had a relative difference median value of 25%, ROI 2 showed a relative difference of 51.5%, ROI 3 11.8%, and ROI 4 had a relative difference median value of 49.5%. The analysis of the lesion SUV_mean_ values showed a mean and median relative difference of 14.4% and 7.6%, respectively, in tracer uptake. The mean uptake value within the lesions for reconstructions by use of MLSS was 9.4 kBq/mL ± 2.9 kBq/mL, whereas the mean lesion uptake for reconstructions utilizing TFSS was 8.1 kBq/mL ± 2.6 kBq/mL.

### 3.2. Cohort 2

**Figure 4:**
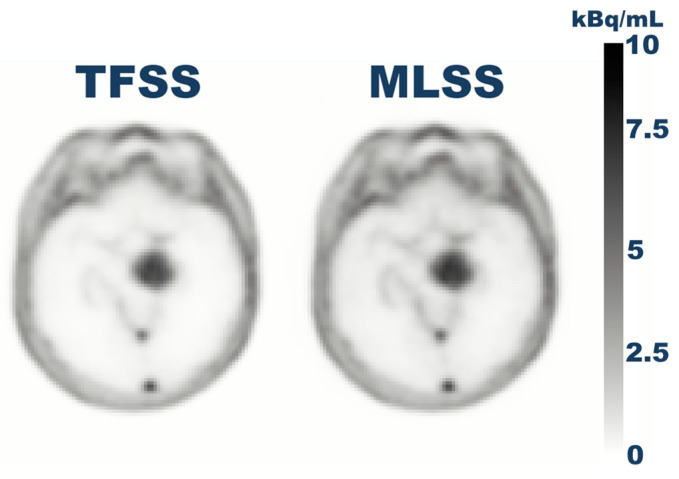
Visualization of TFSS- and MLSS-reconstructed images of a brain scan using [^68^Ga]Ga-uPAR.

Twenty patients were included as PET positive following uptake within the lesion. Correspondingly, 10 patients were included as PET negative with no visible uPAR uptake. Six reconstructions were completed with the use of TFSS for PET-positive patients, along with one completed TFSS reconstruction of a PET-negative patient. All reconstructions were completed with the use of MLSS for both groups. Figure 4 visualizes the TFSS- and MLSS-reconstructed images of a PET brain scan. It is shown that MLSS provides reconstructed images that visually resemble the clinical standard.

The median of the lesion uptake value for MLSS-reconstructed images was 2.6 kBq/mL, and the mean was found to be 2.9 ± 1.2 kBq/mL compared to a median of 2.1 kBq/mL for TFSS-reconstructed images, with a mean of 2.4 ± 1.2 kBq/mL. The investigation of the background uptake within the “positive” group showed a mean of 0.4 ± 0.1 kBq/mL for both groups, indicating no difference in uptake.

The corresponding evaluation of the “background” ROI placed contra-lateral to the lesion or placed within a low uptake area for the PET-negative group showed a range of uptake values between 0.29 and 0.64 kBq/mL for MLSS images and 0.32 kBq/mL for a single successful TFSS image.

## 4. Discussion

The aim of this study was twofold. The study aimed to demonstrate an alternative method for scaling scatter relative to the current state-of-the-art method, TFSS, in cases where it is prone to induce artifacts, such as in proximity to areas with a high tracer uptake contrast. Furthermore, the study investigated whether MLSS was able to provide reconstructed images in scenarios, where it has been observed that TFSS induces termination of the reconstruction process. 

Patients of cohort 1 diagnosed with cervical cancer were included to visualize the difference between the two scatter-scaling methods concerning halo artifacts. The visual investigation of an experienced MD provided the scoring of MLSS reconstruction of 2.89, implying that most of the images were of high image quality. TFSS reconstruction has an average score of 2, indicating that the image was of acceptable image quality (Table 2). However, some possessed the appearance of halo artifacts reducing the score. The TFSS reconstructions of two patient scans showed halo artifacts to a degree, causing a scoring of low image quality. MLSS reconstructions did not show visual halo artifacts. Figure 3 illustrates the relative difference between the reconstructed images by use of TFSS and MLSS. It was found that, in all cases, the uptake value around the bladder showed greater values when MLSS was used compared to TFSS. The relative differences were largest in both lateral positions. The median relative difference in these positions was found to be 51.5% and 49.5%, respectively. Likewise, anteriorly and posteriorly, the findings showed a larger magnitude (25% and 11.8%, respectively) when MLSS was used. This tendency was also found in the investigation of lesion SUV_mean_, with a mean and median relative difference of 14.4% and 7.6%, respectively. Thus, it was found that the image quality of the MLSS reconstructions was evaluated as higher than TFSS reconstructions. Furthermore, the quantification of the surrounding tissue of the bladder showed that MLSS provided higher uptake values and reduced the appearance of halo artifacts.

The uptake values of the TFSS-reconstructed images may be affected if the tail region has low uptake, such as in cases of large patient BMI. Table 1 indicates that one patient was obese, two patients were overweight, and six patients were normal weight. The appearance of halo artifacts within this study was found for both patients with a BMI within the normal range as well as those above the normal range. 

To our knowledge, no studies have yet investigated methods for the removal of halo artifacts on [^68^Ga]Ga-RGD PET. The majority of studies within the area of halo artifacts are evaluated by the use of [^68^Ga]Ga-PSMA-11 PET/MRI [6,14,22] or [^68^Ga]Ga-PSMA-11 PET/CT [7], or by use of [^18^F]FDG PET/CT [10,13]. The critical aspects of halo artifacts appearing within the reconstruction of patient scans with cervical cancer are in correspondence with patients diagnosed with prostate cancer, due to the risk of the halo artifact decreasing lesion contrast. The study by Parker et al. (2020) found that MLSS was able to reduce halo artifacts for [^68^Ga]Ga-PSMA-11 PET/CT scans. Furthermore, the group suggested that MLSS had a favorable quantitative appearance within the pelvic area [7]. This was in correspondence with the findings of our study concerning both the qualitative and quantitative findings, even though the investigations were performed on a PET/CT in the study by Parker et al. (2020) and PET/MRI in this study. The study of Heuβer et al. (2017) found no modality dependence in the appearance of the halo artifact when investigating patients scanned with [^68^Ga]Ga-PSMA-11, which implies that the previous comparisons are assumed valid [14].

A known complication of attenuation correction within the domain of PET/MRI is truncation. The study of Afshar-Oromieh et al. (2017) investigated the effects of arm truncation in [^68^Ga]Ga-PSMA-11 PET/MRI scans relative to the observation of halo artifacts. Their method was to either scan the patient’s arms down or arms elevated above their heads. The findings showed a significant reduction in the appearance of halo artifacts if the arms were elevated, highlighting the importance of the attenuation correction of the PET image [31]. In this study, an MRI-based synthetic generated CT attenuation correction map was used during the reconstruction of the patient scans within cohort 1. It was shown by Ahangari et al. (2021) that the sCT was acceptable for PET attenuation correction and radiotherapy-planning dose calculations [26]. Furthermore, the patients were scanned with the arms outside the FOV, which led to the assumption that the halo artifacts found within the patients of cohort 1 were not related to truncation artifacts. The attenuation correction of the patients of cohort 2 was also based on an MRI-derived deep-learning-based attenuation correction map by Ladefoged et al. (2019) to compute attenuation correction maps [28]. Furthermore, the patients were scanned with their arms along the body outside the FOV of the brain region. Thus, it was also assumed that the attenuation correction of all the PET/MRI scans included in this study was performed by use of the best possible method concerning attenuation correction.

The aim of including cohort 2 in the investigation of the scatter-scaling methods was to evaluate whether MLSS was a viable option for replacement of the clinical standard, TFSS, in cases of low background uptake, such as in scans with [^68^Ga]Ga-uPAR, where reconstruction abortion has been observed within the clinical routine. The resulting investigations showed that the reconstructions were aborted in 23 out of 30 scans due to the non-negativity constraint of TFSS for the scanners used in the current study. MLSS does not possess non-negativity constraints, causing it to abort the reconstruction. The reconstruction by use of MLSS reconstruction provided images for all patient scans suggesting that MLSS can provide patient images for patient scans which TFSS reconstruction was not able to. The uptake value found in the reconstructed image by use of either MLSS or TFSS showed similar values, as visualized in Figure 4. This suggests that MLSS and TFSS produce reconstructions of similar quality in these cases. The mean lesion uptake value for the MLSS-reconstructed images was 2.9 ± 1.2 kBq/mL compared to the mean lesion uptake within the TFSS-reconstructed images, which was 2.4 ± 1.2 kBq/mL. This indicates that the lesion uptake values were higher for MLSS-reconstructed images. However, the difference is not significant. The investigation of the background uptake resulted in the same mean values by use of each method, implying that the background uptake did not show any significant difference. The findings by use of cohort 2 concluded that MLSS can robustly provide reconstructed images in scenarios where TFSS cannot. However, in the cases where TFSS provides successful reconstructions, there was no significant difference between the methods, implying that MLSS provided uptake values that resembled the clinical standard.

Currently, when TFSS induces artifacts or does not provide reconstructed images, the clinical scatter fallback method is ABS in our clinic. The scatter estimation by ABS is not iterative and often leads to a biased quantification. This was elaborated in the study of Bal et al. (2021) through a phantom study. The investigation of the uptake values of the phantom illustrated that ABS provided too large of SUVs due to an underestimation of scatter, whereas the uptake values of the reconstructed image by use of MLSS were close to the expected SUV of 1.00 [13]. Our study did not evaluate the MLSS relative to ABS due to ABS being considered as a fallback method. The aim was to compare the method relative to the clinical standard and evaluate whether MLSS can perform in correspondence to the standard or perform superiorly to the standard relative to artifacts. 

The limitation of this study was the lack of a phantom study to support the patient investigation. Since no ground truth is known, the comparison was performed as a relative difference. It is believed that a relative comparison between the methods provides valuable information about the performance of the methods within areas prone to halo artifacts, or whether the reconstructions failed. The findings of this study showed larger differences between the method of use compared to the findings of the phantom study of Bal et al. (2021), which may be due to the study of Bal et al. (2021) being performed with the use of a [^18^F]FDG tracer rather than the [^68^Ga]-labeled tracer. Another limitation of this study was the size of the cohorts. Cohort 1 included only nine patients, whereas the quantitative investigation of cohort 2 included only six patients. However, cohort 2 highlighted the observed problem by the termination of the reconstruction process using TFSS through the limited number of completed reconstructions. The size of the cohorts was inadequate to perform reliable statistical investigations. 

Overall, it was found that MLSS reconstruction is a robust method of use concerning both qualitative and quantitative assessment. Furthermore, the MLSS can be used for the retrospective study of patient scans showing severe halo artifacts. Future work regarding the investigation of the robustness performance of MLSS includes a comparison of TFSS and MLSS in whole-body [^18^F]FDG and [^68^Ga]Ga-PSMA-11 PET/CT.

## 5. Conclusions

MLSS has shown promising results for the reduction of halo artifacts and for use in high-contrast images in which TFSS reconstruction introduces halo artifacts. The blinded qualitative evaluation of MLSS-reconstructed images had an average score of 2.98 compared to the TFSS reconstructions, with a score of 2. MLSS reconstructions successfully provided image reconstructions in scenarios whereas TFSS reconstructions were found to fail in 23 out of 30 patient scans of cohort 2. The investigation of the lesion uptake of cohort 2 showed that MLSS and TFSS reconstructions showed uptake values within a similar range. It was found that the successfully provided reconstructions could not be found different. The study was limited by a lack of ground truth or a comparison of the methods through a phantom study. Based on this study, it can be suggested that MLSS can replace TFSS within the reconstruction procedure.

## Figures and Tables

**Table 1:** Overview of age, weight, height, and BMI of the patients included in cohort 1.

Patient	Age (Years)	Weight (kg)	Height (m)	BMI
1	68	84	1.61	32.4
2	29	54	1.61	20.8
3	65	76	1.70	26.3
4	68	78	1.73	26.0
5	45	60	1.68	21.3
6	60	70	1.79	21.9
7	55	69	1.71	23.6
8	46	65	1.78	20.5
9	74	54	1.55	22.5

**Table 2:** Overview of the qualitative assessment score. The scoring system is given by 1 = low image quality, 2 = acceptable image quality, and 3 = high image quality.

Method	Score = 1	Score = 2	Score = 3
MLSS	0	1	8
TFSS	2	5	2

## Data Availability

Data supporting the reported results can be obtained via contact with the corresponding author upon reasonable request and legal approval. The data are not publicly available due to a no public data sharing agreement.
